# Mechanical Properties of Smart Polypropylene Meshes: Effects of Mesh Architecture, Plasma Treatment, Thermosensitive Coating, and Sterilization Process

**DOI:** 10.1021/acsbiomaterials.3c00311

**Published:** 2023-05-26

**Authors:** Sonia Lanzalaco, Christine Weis, Kamelia A. Traeger, Pau Turon, Carlos Alemán, Elaine Armelin

**Affiliations:** † IMEM-BRT Group, Departament d’Enginyeria Química, EEBE, Universitat Politècnica de Catalunya, C/Eduard Maristany, 10-14, 08019 Barcelona, Spain; ‡ Barcelona Research Center in Multiscale Science and Engineering, Universitat Politècnica de Catalunya, 08930 Barcelona, Spain; § Research and Development Centre, B. Braun Surgical, S.A.U., Carretera de Terrassa 121, Rubí, Barcelona 08191, Spain; ∥ Institute for Bioengineering of Catalonia (IBEC), The Barcelona Institute of Science and Technology, Baldiri Reixac 10-12, 08028 Barcelona, Spain

**Keywords:** biomedical implant, bursting test, EtOx sterilization, poly(*N*-isopropylacrylamide), pull out test, surgical mesh

## Abstract

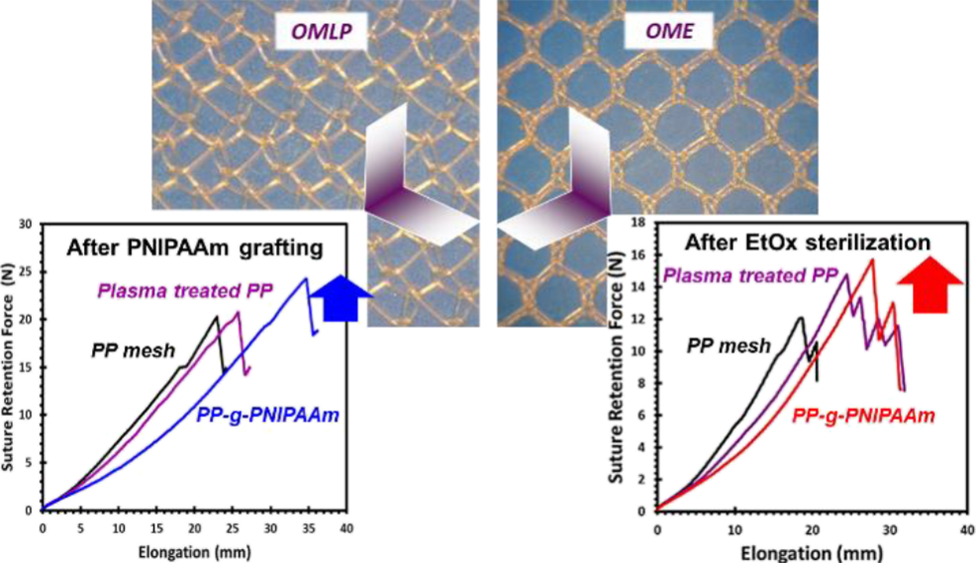

Smart polypropylene (PP) hernia meshes were proposed to detect surgical infections and to regulate cell attachment-modulated properties. For this purpose, lightweight and midweight meshes were modified by applying a plasma treatment for subsequent grafting of a thermosensitive hydrogel, poly(*N*-isopropylacrylamide) (PNIPAAm). However, both the physical treatment with plasma and the chemical processes required for the covalent incorporation of PNIPAAm can modify the mechanical properties of the mesh and thus have an influence in hernia repair procedures. In this work, the mechanical performance of plasma-treated and hydrogel-grafted meshes preheated at 37 °C has been compared with standard meshes using bursting and the suture pull out tests. Furthermore, the influence of the mesh architecture, the amount of grafted hydrogel, and the sterilization process on such properties have been examined. Results reveal that although the plasma treatment reduces the bursting and suture pull out forces, the thermosensitive hydrogel improves the mechanical resistance of the meshes. Moreover, the mechanical performance of the meshes coated with the PNIPAAm hydrogel is not influenced by ethylene oxide gas sterilization. Micrographs of the broken meshes evidence the role of the hydrogel as reinforcing coating for the PP filaments. Overall, results confirm that the modification of PP medical textiles with a biocompatible thermosensitive hydrogel do not affect, and even improve, the mechanical requirements necessary for the implantation of these prostheses in vivo.

## Introduction

More than 20 million of hernias are annually repaired worldwide. Among the different types of hernias, the incidence of inguinal hernia is the highest, accounting for 75% of the cases.^1,2^ Within this context, the unceasing research pushed by industry, professional societies and academy has played a major role bringing scientific and technological progress and enabling the development of more adaptable materials.^3,4^ Currently, there are meshes with different knitting structures in the market,^5^ which enhance postoperative rehabilitation quality for patients and lower the recurrence rate.^4,6^ Although the surgical mesh market is segmented on the basis of the used material, nowadays, the most ubiquitous are based on nonabsorbable polymers.^3,7,8^ Among them, polypropylene (PP) is advantageous due to its nontoxicity, high biocompatibility, and minimized contamination during processing.^9^

The selection of the mesh is carried out by surgeons for each patient, considering the kind of defect to be repaired, which is crucial to avoid or minimize postoperative complications and recurrences. Despite this, postsurgical problems such as mesh shrinking and foreign body reactions leading to inflammatory processes are complications associated with such biomedical materials.^10,11^ On the other hand, more than 10% of patients are likely to be rehospitalized due to adhesion-related complications within 5 years of surgery,^12^ and 2 to 8% of implanted meshes might become infected.^13−15^ To mitigate such adverse consequences, many research efforts have been focused on altering the surface properties of synthetic meshes, either directly^16^ or through the application of antiadhesion films,^17^ antibiotic coatings, or fibers loaded with silver zeolites.^18^ Also, prevention strategies, such as the incorporation of a sensor to early alert about bacterial growth,^19^ have been reported.

In recent work, the plasma technique was employed to modify the surface polymer prostheses based on the PP matrix for incorporating a coating of the poly(*N*-isopropylacrylamide) (PNIPAAm) hydrogel.^4,2−23^ This strategy provided meshes with enhanced cell attachment and detachment modulated properties.^22,23^ More recently, Learn et al.^24^ also demonstrated the advantageous modification of PP meshes with cold plasma, showing that such treatment reduces fibrinogen absorption and bacteria attachment, which are both intrinsically related to the surface oxygen content.

Several synthetic materials different from PP have been proposed to manufacture surgical meshes bringing new physical and mechanical properties to minimize hernia recurrence (i.e., resorption, flexibility, and degradation profile).^25^ In practice, mesh mechanical properties and tissue integration are influenced by several factors, such as material nature, mesh porosity, mesh weight, and fabrication method, among others.^26−28^ Moreover, medical biotextiles must overcome the conditions and methods used for sterilization, which could affect the mechanical performance of the implant. Ethylene oxide (EtOx) gas is one of the most common methods to sterilize materials and devices in the healthcare sector, becoming the only alternative when other sterilization methods cannot be employed (i.e., heat and radiation) despite its intrinsic risks (i.e., explosion, environmental, and health if residual products remain in the medical device after sterilization). Nevertheless, despite alternatives to EtOx sterilization are intensively searched and desired, it still shows some significant advantages, such as a highly efficient decontaminating process, its low impact on material properties, and its relatively low cost.^29^

In this work, we focus on the impact of a thermosensitive hydrogel (PNIPAAm) on the mechanical properties of a knitted surgical mesh of PP intended to develop a new generation of smart meshes responsive to stimulus. In the resulting functionalized PP-*g*-PNIPAAm meshes, the hydrogel acted as an adaptable and thermally responsive coating.^4^ The impact of the mesh architecture on the morphology of the coating and the mechanical properties of the implant have been examined considering two different meshes, which differ in weight per area, pores size, and directional elasticity. In addition, since medical devices must be sterilized before implantation in the human body, the effect of the sterilization process on the properties of this new generation of meshes has also been analyzed. For this purpose, the EtOx sterilization method, which is currently used for the commercial nonfunctionalized (pristine) PP meshes was applied. Overall, results show that plasma treatment used prior to grafting the hydrogel affects negatively the bursting and suture pull out force, whereas after grafting, the thin layer of thermosensitive PNIPAAm becomes beneficial, enhancing both the mechanical forces and the elongation at break. In addition, the sterilization with EtOx gas induced the behavior of PNPAAm as a plasticizer of the PP yarns, improving the overall resistance of the material to rupture.

## Methods

### Materials

Monofilament PP meshes, which were provided by B. Braun Surgical S.A.U. (Rubí, Spain), were used in this work. More specifically, meshes with two different architectures were considered: Optilene mesh LP (OMLP) and Optilene mesh elastic (OME). OMLP is a lightweight (36 g/m^2^) mesh with 0.39 mm of thickness and 1 mm of pore diameter, while OME is a midweight (48 g/m^2^) mesh, 0.55 mm of thickness and 3.6 × 2.8 mm^2^ pore size. Both, OMLP and OME, are flexible and nonabsorbable meshes.

*N*-isopropylacrylamide (NIPAAm; 99%, CAS 2210-25-5), *N*,*N*′-methylene-bisacrylamide (MBA; 99%, CAS 110-26-9), and *N*,*N*,*N*′,*N*′-tetramethylethylenediamine (TEMED, 99%, CAS110-18-9) were purchased from Sigma-Aldrich (Spain). Ammonium persulfate (APS; 98%, CAS7727-54-0) was supplied by Panreac S.A. All reagents were used as received. Milli-Q water grade (0.055 S/cm) was used in all synthetic processes used to graft the thermosensitive hydrogel onto the mesh filaments. Nitrogen used was of pure grade (99.995%).

### PP-*g*-PNIPAAm Mesh Preparation

PP meshes were functionalized using a previously reported procedure.^22,23^ Briefly, this was performed in two successive steps. For the first step, OMLP and OME meshes were put within plasma equipment and the whole system was purged under vacuum and filled with oxygen gas. After this, the system was evacuated until the desired pumping down pressure, which was 0.03 mbar. The mesh surface was irradiated employing an LFG generator 1000 (Diener Electronic GmbH Co., Germany) using a plasma power of 250 W during 180 s. After the plasma treatment, all samples were stored under vacuum for a few days, if not used immediately.^23^ This plasma irradiation treatment allowed forming polymer radicals on the PP surface meshes.

After plasma surface modification of the PP meshes, the grafting of the PNIPAAm hydrogel was performed using the conditions reported in a former work.^22^ Thus, graft copolymerization of the NIPAAm monomer onto the meshes treated with low-pressure oxygen plasma was performed as follows. The NIPAAm monomer (0.5658 g, 250 mM), MBA cross-linker (0.0031 g, 1 mM), and TEMED accelerator (0.0065 g, 2.77 mM) were dissolved in 20 mL of water in a reaction vessel. After total dissolution, reagents were mixed with the meshes in the same reaction vessel. The solution was stirred, and nitrogen gas flow was bubbled through for 30 min to remove dissolved oxygen before the addition of the catalyst. Then, 0.15 mL of APS (370 mM) aqueous solution was added to the vessel to initiate the polymerization. The temperature was maintained at 30 °C with a water bath. After 2 h of reaction, the PNIPAAm-coated meshes were extracted and poured onto 400 mL of deionized water, stirring for 4 h for purification. The resulting meshes, hereafter denoted OME-*g*-PNIPAAm and OMLP-*g*-PNIPAAm depending on the architecture of the mesh, were dried at 30 °C overnight under vacuum.

The surface weight of the hydrogel onto PP meshes obtained using a grafting time of 2 h, which was observed to provide good sensitivity in thermal response while maintaining cell attachment-modulated properties,^4,21−23^ was 112.9 ± 22.3 and 104.4 ± 21.6 g/m^2^ for OME-*g*-PNIPAAm and OMLP-*g*-PNIPAAm, respectively. Other conditions used exceptionally for specific tests on meshes with higher surface weight are described in the Results and Discussion section. In order to examine the effect of the amount of hydrogel grafted onto the hydrogel, a longer reaction time (4 h) was used for specific tests on OME, with a final surface weight of 185.2 ± 6.2 g/m^2^.

### Mesh Characterization

Raman spectra were acquired using a Renishaw dispersive Raman microscope spectrometer (model InVia Qontor, GmbH, Germany) and Renishaw WiRE software. The spectrometer is equipped with a Leica DM2700 M optical microscope, a thermoelectrically cooled charge-coupled device (CCD) detector (−70 °C, 1024 × 256 pixels), and a spectrograph scattered light with a 2400 lines/mm of grating. The experiments were performed with a 532 nm excitation wavelength and with a nominal laser power between 1 and 100 mW output power. The exposure time was 10 s, the laser power was adjusted to 1% of its nominal output power and each spectrum was collected with three accumulations. All Raman spectra were collected in a spectral range from 600 to 4000 cm^–1^ with the same measurement parameters.

Scanning electron microscopy (SEM) analyses were conducted using a focused ion beam Zeiss NEON40 scanning electron microscope equipped with an energy-dispersive X-ray analysis (EDX) spectroscopy system and operating at 5 kV. SEM was used to examine the surface morphology of the PP-*g*-PNIPAAm filaments before and after sterilization. For this purpose, the meshes were mounted on a double-side adhesive carbon disk and sputter-coated with a thin layer of carbon to prevent sample charging problems.

X-ray photoelectron spectroscopy (XPS) analyses were used to confirm plasma activation and PNIPAAm gel grafting. Samples were supported on aluminum substrates. The assays were performed on a SPECS system equipped with an Al anode XR50 source operating at 150 mW and a Phoibos MCD-9 detector. The pressure in the analysis chamber was always below 10^–7^ Pa. The pass energy of the hemispherical analyzer was set at 25 eV, and the energy step was set at 0.1 eV. Data processing was performed with the CasaXPS program (Casa Software Ltd., UK).

Before each mechanical test, samples were weighted in a Sartorius Analytical balance (model Secura 125-1S) and the thickness of PP filaments was measured with a Micrometer Mitutoyo (model C112XB). The thickness was determined in five points of the mesh, corresponding to the middle and laterals zones of the sample surface. For each sample, the surface weight (in g/m^2^) was plotted against the filament thicknesses (in mm) to analyze the effect of hydrogel density (i.e., the amount of hydrogel per unit of area) on the mechanical behavior.

### Biocompatibility Studies

MCF-7 cells (epithelial cells) and COS-1 cells (fibroblast cells) were selected for biocompatibility assays due to their rapid growth. Cells were cultured in Dulbecco’s Modified Eagle Medium (DMEM, 4500 mg/L of glucose) supplemented with streptomycin (100 μg/mL), penicillin (100 units/mL), l-glutamine (2 mM), and fetal calf serum (FBS; 10%). Cell cultures were maintained in a humidified incubator with an atmosphere of 5% CO_2_ and 95% O_2_ at 37 °C. Culture media were changed every 2 days. When the cells reached 80–90% confluence, they were detached using 2 mL of trypsin (0.25% trypsin/EDTA) for 5 min at 37 °C. Finally, cells were resuspended in 5 mL of fresh medium and their concentration was determined by counting with a Neubauer camera using 0.4% trypan blue as a vital dye.

Biocompatibility studies were performed on untreated, plasma-treated, and functionalized OMLP samples with an area of 1 × 1 cm^2^, which were fixed in stainless steel substrates (to prevent the samples from floating in the culture medium). Then, fixed samples were placed in polystyrene plates of 24 wells and were sterilized using UV irradiation for 15 min in a laminar flux cabinet. Controls were performed for culturing cells on the stainless steel substrates used to fix the samples but without any kind of PP meshes. Assays were performed by seeding 5 × 10^4^ of cells on the surface of the sample placed in each well. The attachment of cells to the mesh surface was promoted by incubating under culture conditions for 30 min. Finally, 2 mL of the culture medium was added to each well. Cells in the well were quantified after 24 h to evaluate their adhesion to the untreated, plasma-treated, and functionalized meshes. Cultured cells were again quantified after 7 days to evaluate the biocompatibility of the samples. The number of viable cells was evaluated by the colorimetric MTT [3-(4, 5-dimethylthiazol-2-yl)-2,5-diphenyltetrazolium bromide] assay.^30^ The viability was expressed as a relative percentage referred to the number of cells in the control (i.e., a stainless steel substrate without the mesh).

Assays were performed using four replicates and results were averaged. The statistical analysis was performed by the one-way ANOVA test to compare.

the means of all groups. The *t*-test was applied to determine a statistically significant difference between different groups. The tests were performed with a confidence level of 95% (*p* < 0.05).

### Mechanical Testing

#### Material Preparation

Before mechanical assays, meshes were previously wet by immersion in distilled water during 15 min at a temperature of 37 °C. After this, samples were extracted and subjected to bursting and suture retention tests out of the solution, allowing to evaluate the mechanical properties with the amount of water absorbed at the desired conditions.

#### Bursting Tests of PP-Based Meshes

The bursting strength test, which is used to determine the maximum break strain of meshes under compression forces, was performed according to the ASTM method D-3787.^31^ Assays were conducted using Zwick equipment (Model Z005) with a 5 kN load cell at a constant rate of 50 mm/min and a distance between the pendulum and the material of 0 mm. Samples were prepared by cutting the meshes in specimens of 80 × 80 mm^2^. Five samples were tested for each of the following conditions: (a) untreated meshes (OME and OMLP); (b) low-pressure O_2_ plasma-treated meshes (OME plasma-treated and OMLP plasma-treated); and (c) PP-*g*-PNIPAAm meshes (OME-*g*-PNIPAAm and OMLP-*g*-PNIPAAm). The breaking force (N) and the elongation at failure (mm) were measured to evaluate alterations in the mechanical properties of the modified samples with respect to the untreated ones.

#### Suture Pull Out Tests of PP-Based Meshes

The suture pull out test, which is used to determine the suture tearing out resistance of surgical knitted meshes with suture threads, is a crucial test for the final implantation of the mesh inside the human body, proving the effective fixation of the implant. Moreover, the suture pull out strength is recommended in the literature to be 20 N.^32^ The suture pull out strength of the mesh must be greater than this value to ensure safe fixation. Therefore, centered points were marked at a distance of 2 mm to the longer cutting edge, while, for the latter, the marking was in the third undamaged knitting loop (counted from the cutting edge) in the center of the longitudinal cut on each side. After the marking, the samples were penetrated at the marked points with a polypropylene USP 3/0 HR suture. The sample size was about 30 × 45 mm^2^ in surface area, after cutting them by a pair of scissors. The cut edge of the specimen was placed parallel to the jaw face into the lower grip and the suture was tightened into the upper grip. The testing speed was 100 mm/min, and the maximum tear out force was recorded for five different specimens.

#### Tensile Strain Tests

Strain–stress curves were obtained with a Zwick Z2.5/TN1S testing machine equipped with a temperature chamber and with integrated testing software (testXpert, Zwick). The deformation rate for stress–strain assays was 1 mm/min.

### Sterilization with Ethylene Oxide (EtOx)

#### Sterilization Method

The sterilization of the meshes without and with grafted hydrogels was performed using the low temperature EtOx cycle (40 °C) in Suphatec S.L. equipment available at B Braun Surgical S.A.U. Samples were sterilized in the presence of EtOx gas during 540 min at a temperature in the range of 37–43 °C. The process fulfills the UNE-EN ISO 11135-2015 standard for sterilization processes.^33^

#### Sterility Test

The sterility test was carried out following other standard: ISO 11737-2:2009.^34^ Three replicates of each sample were introduced inside bottles with Tryptone Soya Broth (TSB) medium under sterile conditions. The bottles were incubated for 7 days at 20–25 °C and another 7 days at 30–35 °C to verify their sterility. Sterility test results were obtained from the visual examination of culture bottles.

#### EtOx Residue Evaluation

The evaluation of the residues coming from the sterilization process, mainly based on EtOx and ethylene chlorohydrin traces, was performed at Echevarne Laboratory (Barcelona, Spain) by means of the gas chromatography-flame ionization detector (GC-FID)/head space (GC-FID/HS) technique and following the ISO10993-7/09: UNE-EN ISO 10993-7:2009/AC:2010 procedure.^35^

### Statistical Evaluation

Statistical statement of the mechanical properties tests was carried out following the ISO rules reported in the corresponding sections. In all cases, five specimens were used and the average value and standard deviation reported. The sterility tests were carried out in triplicate. However, no statistical evaluation was performed since no microbial growth was observed (number of colony units equal to zero) in any of the sterilized samples.

## Results and Discussion

### Characterization of the Functionalized Surgical Meshes

**Figure 1:**
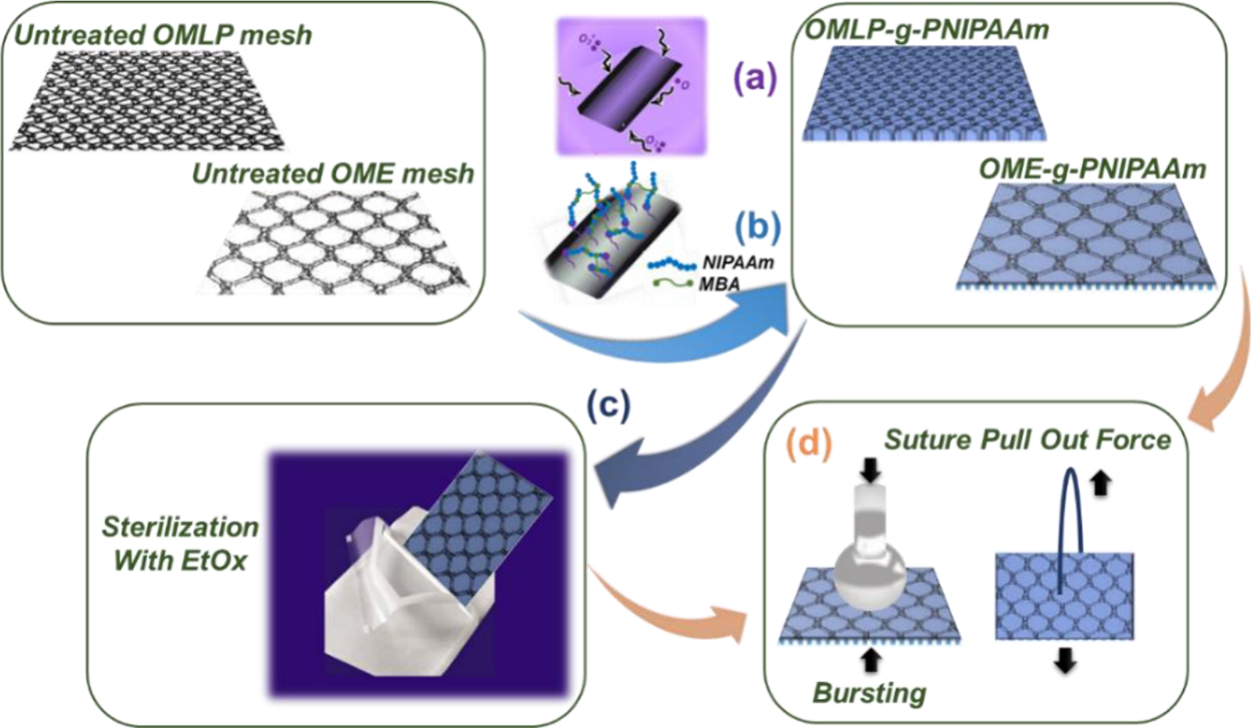
Sketch showing the aim of this study: (a) low-pressure O_2_ plasma functionalization of lightweight and midweight PP meshes; (b) grafting with NIPAAm-*co*-MBA monomers; (c) sterilization with EtOx gas; and (d) mechanical tests used to evaluate the properties of the modified and sterilized meshes.

Figure 1 summarizes the experimental work carried out in this study, which mainly consists of evaluating the mechanical properties of modified PP surgical meshes and unmodified OMLP (light density) and OME (medium density) meshes. More specifically, the prostheses were modified considering the following three-steps: (a) functionalization by applying a low-pressure O_2_ plasma treatment; (b) coating by grafting the biocompatible hydrogel; and (c) sterilization with EtOx. On the other hand, evaluation of the mechanical properties of pristine and modified meshes (i.e., functionalized, grafted, and sterilized) was performed using bursting and suture retention assays. The setup and equipment used for the mechanical assays is displayed in Figure S1.

**Figure 2:**
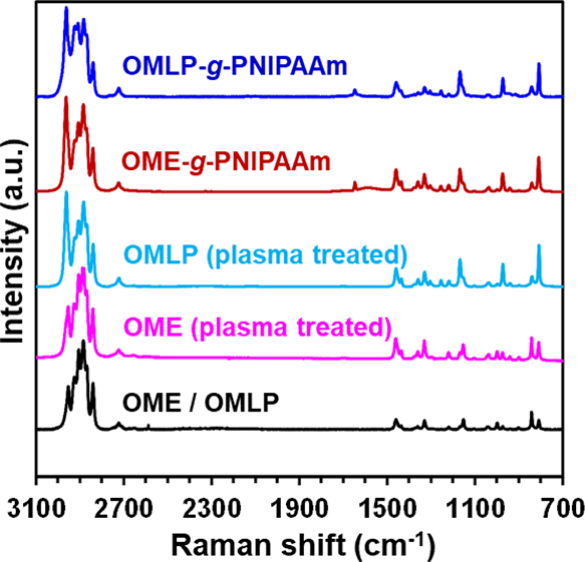
Raman spectra of untreated meshes (OME/OMLP), plasma-treated meshes, and hydrogel-grafted specimens (OME-*g*-PNIPAAm and OMLP-g-PNIPAAm).

The Raman spectra of untreated (pristine sample), plasma-modified, and hydrogel-coated OME and OMLP are compared in Figure 2, while the main absorption bands are summarized in Table 1. As it was expected, the spectra of the two pristine meshes are practically identical and display the typical bands of PP.^36,37^ The low-pressure oxygen plasma treatment affects the intensity of the CH_3_ groups, increasing the intensity of the bands at 809, 973, and especially, 2915 cm^–1^, which is in agreement with previous observations.^38^ Indeed, comparison of the Raman spectra recorded for the two plasma-treated meshes reveals that such effect is more pronounced for OMLP than OME, which is in agreement with previous observations that showed how the results of physical and chemical treatments on PP surgical meshes are effected by the complexity of their geometry.^19,38^ The grafted PNIPAAm hydrogel in OME-*g*-PNIPAAm and OMLP-*g*-PNIPAAm was detected by the presence of the amide I band at 1647 cm^–1^.

**Table 1:** Main Raman Fingerprints of the Untreated, Plasma-Functionalized, and Hydrogel-Grafted OME and OMLP Meshes Studied in This Work

sample	band
pristine OME and OMLP	• 809 and 973 cm^–1^ (vibrations of the CH_3_ groups from crystalline domains)
	• 841 cm^–1^ (rocking of CH_3_ groups from amorphous domains)
	• 999, 1167, and 1455 cm^–1^ (CH_2_ unsaturation and deformation vibrations)
	• 1150 cm^–1^ (C–C stretching and CH bending)
	• 1330 cm^–1^ (C–H stretching, CH_2_ wagging, and CH_3_ bending)
	• 2918 cm^–1^ (C–H stretching from CH_2_)
	• 2953 cm^–1^ (C–H stretching from CH_3_)
low-pressure O_2_ plasma-treated OME and OMLP	• increment of the relative intensity of the bands associated to CH_3_ groups at 809, 973, and 2953 cm^–1^
OME-*g*-PNIPAAm and OMLP-*g*-PNIPAAm	• appearance of the amide I band at 1647 cm^–1^

To further confirm the success of the plasma treatment and the PNIPAAm grafting, XPS analyses were performed on OMLP, plasma-treated OMLP, and OMLP-*g*-PNIPAAm meshes. Results are displayed in Table 2, which also lists the O/C, N/C, and N/O ratios. Atomic compositions were estimated subtracting the concentration of C and O detected in an aluminum holder. As it was expected, the atomic concentration of O 1s increased noticeably after the plasma treatment, while the hydrogel grafting was confirmed by the appearance of a significant amount of N 1s.

**Table 2:** Atomic Concentration (in %) of C 1s, O 1s, and N 1s for Untreated OMLP, Plasma-Treated OMLP, and OMLP-*g*-PNIPAAm Meshes; the O/C, N/C, and N/O Ratios Are Also Displayed for Comparison

sample	element	atomic conc. (%)	O/C	N/C	N/O
OMLP	C 1s	98.28			
	O 1s	1.72	0.02		
	N 1s				
plasma-treated OMLP	C 1s	74.46			
	O 1s	25.54	0.34		
	N 1s				
OMLP-*g*-PNIPAAm	C 1s	77.49			
	O 1s	13.78	0.18	0.11	0.63
	N 1s	8.73			

**Figure 3:**
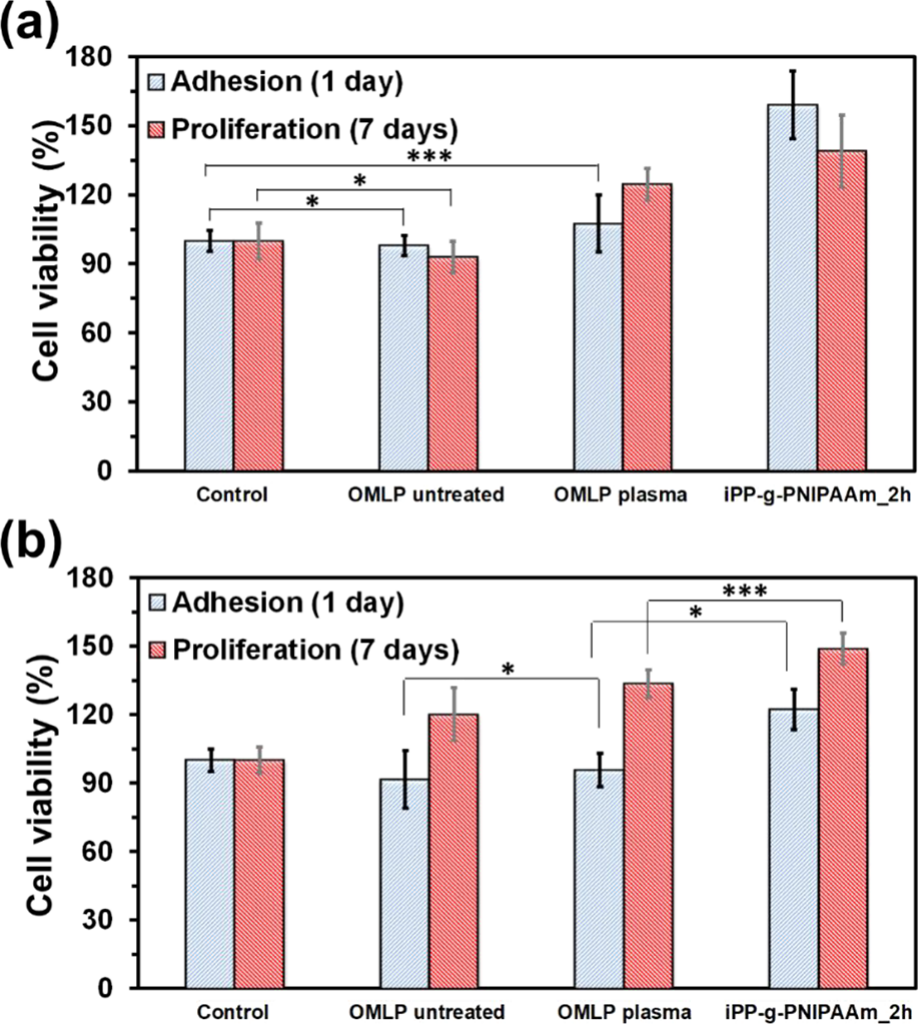
Cellular adhesion and cellular proliferation on the surface of untreated OMLP, plasma-treated OMLP and OMLP-*g*-PNIPAAm. Stainless steel was used as a control substrate. (a) MCF-7 and (b) COS-1 cells were cultured during 24 h and 7 days. Asterisk marks (*) and (***) represent significant difference among the samples at *p* < 0.05 and *p* < 0.001, respectively.

The effect of the plasma treatment and the hydrogel in the biocompatibility of PP meshes was evaluated by examining cell adhesion and cell proliferation using two cell lines with fast growth. These are MCF-7 and COS-1, which are epithelial and fibroblast cells, respectively. Assays were performed using untreated OMLP, low-pressure O_2_ plasma-treated OMLP, and OMLP-*g*-PNIPAAm, and stainless steel used to avoid the flotation of such samples on the culture medium (see the Methods section) being the control. Quantitative results for cell adhesion and proliferation assays (24 h and 7 days of cell culture, respectively) are displayed in Figure 3. Results, which correspond to the average of four independent replicas for each system, are expressed in terms of cell viability relative to the control.

The amount of cells adhered to pristine and plasma-treated PP meshes is similar to that of steel control, which is a well-known biocompatible material. This behavior improves after the functionalization with the hydrogel, indicating that PNIPAAm has a major impact on the interaction and attachment of the cells to the surface. This has been attributed to the hydrophilic nature and 3D structure induced by the grafted hydrogel, as was observed in OMLP-*g*-PNIPAAm samples.^4,23^ Thus, such parameters favor the adhesion of the cell, facilitating the interaction with filopodia filaments in cells, which are thin actin-rich structures protruding from the lamellipodial actin network that play a crucial in cell adhesion.^39,40^

Results for cell proliferation were also independent of the cell line. Proliferation of cells on plasma-treated OMLP was better than on pristine meshes, indicating that the species created by the low-pressure O_2_ plasma treatment favor cell division (cytokinesis). However, OMLP-*g*-PNIPAAm samples showed the highest cell viability after 7 days, evidencing that the hydrogel promotes cell growth. More specifically, the PNIPAm hydrogel improved the cell proliferation with respect to pristine OMLP by a factor of 1.4 and 1.2 for MCF-7 and COS-1 cells, respectively. Thus, the grafting of the hydrogel on the PP meshes promotes considerable cell adhesion and growing.

The swelling property of hydrogels affects significantly their mechanical properties. In the case of PNIPAAm grafted to the studied meshes, such property was examined in previous work.^22^ It was shown that the swelling ratio for water observed at 25 °C, 25.5% ± 3.4%, decreased to 10.6% ± 1.4% at 37 °C, which is the temperature used to prepare the samples for mechanical assays. Comparison of the swelling ratio obtained using a phosphate buffer saline (PBS) solution (26.7% ± 3.2 and 11.7% ± 0.9% at 25 and 37 °C, respectively) revealed that the solvent does affect the swelling property of PNIPAAm. Accordingly, although samples were heated to the physiological temperature for mechanical tests, water was maintained as solvent.

Finally, before starting mechanical studies, the surface weight and filament thickness of untreated, plasma-treated, and grafted meshes were determined. Results, which are listed in Table 3 indicate that the low-pressure O_2_ plasma slightly affects both the surface weight and the thickness of the filaments decreasing them, even though such reduction was very small (1–2 and 6–8%, respectively). Conversely, the grafting with the hydrogel drastically increases both the surface weight and the filament thickness (by a factor of ∼2.5 and ∼1.3, respectively).

**Table 3:** Effect of Plasma Treatment and Grafting of the PNIPAAm Hydrogel on the Surface Weight and Thickness of OME and OMLP Meshes

sample	surface weight (g/m^2^)	filament thickness (mm)
OME untreated	52.09 ± 1.19	0.756 ± 0.007
OMLP untreated	36.87 ± 0.90	0.388 ± 0.007
OME plasma-treated	51.07 ± 0.75	0.711 ± 0.015
OMLP plasma-treated	36.54 ± 0.42	0.356 ± 0.015
OME-*g*-PNIPAAm	112.89 ± 22.27	0.954 ± 0.211
OMLP-*g*-PNIPAAm	104.45 ± 21.64	0.498 ± 0.092
OMLP-*g*-PNIPAAm sterilized	103.61 ± 11.52	0.536 ± 0.040

### Bursting Properties of Untreated, Plasma-Treated, and Coated Meshes

Although the mechanical properties of untreated PP surgical meshes have been previously reported,^41,42^ the application of treatments to improve the healing process and reduce hernia recurrence (e.g., chemical etching^43^ and coating with another biocompatible substance^16,44^) affect the suture resistance and burst forces, which influence the final application of such biomedical devices.^45^ The bursting test, which evaluates the tensile strength of constrained meshes subjected to a perpendicular force, examines the ability of the implants to withstand biaxial loading that may be encountered during changes in intra-abdominal pressure in vivo. In this section, the effect of the plasma treatment and PNIPAAm grafting on the bursting strength of OME and OMLP meshes are analyzed. All assays were performed using samples preheated at 37 °C by immersion in water at such temperature during 15 min.

**Figure 4:**
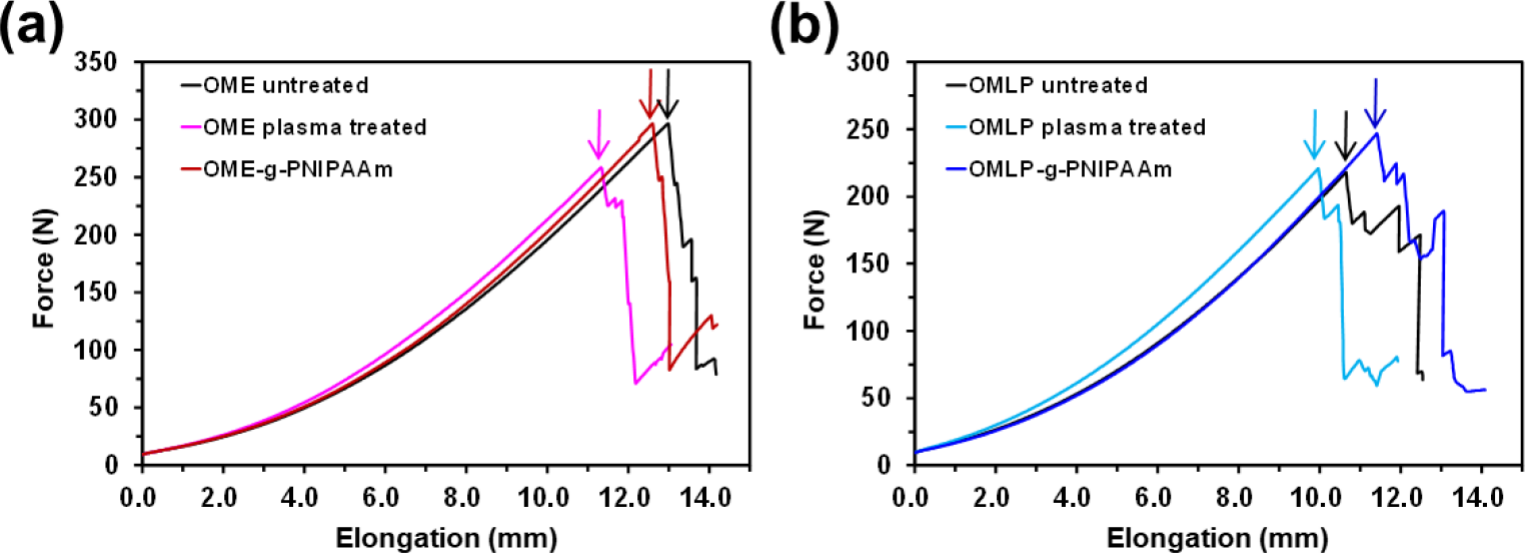
Effect of the plasma treatment and PNIPAAm grafting on the bursting properties of (a) OME and (b) OMLP meshes. Arrows indicate the maximum strength values.

The force–elongation curves obtained for pristine and modified OME and OMLP meshes are displayed in Figure 4, while the bursting properties are summarized in Table 4. Plasma treatment produced some moderate changes in the burst force and the elongation to failure. The highest reduction was observed for the elastic mesh (OME), which exhibited a loss of 14% in force and of 11% in elongation at break (Table 4). In the case of the lightweight density mesh (OMLP), the burst force was kept almost constant after plasma treatment (218.2 ± 15.00 N vs 218.9 ± 12.70 N) while the elongation at break decreased 7% only. Such small reductions were attributed to plasma-induced changes in the PP chains. Learn et al.^24^ showed that long plasma treatments cause some embrittlement of PP yarns and affect their mechanical properties, which was attributed to the fact that oxygen-rich functionalities are probably created via scission of the polymer chain at the surface of the treated specimens. These imperfections, which behave as very small sites for crack nucleation, increase with the time of exposure to the plasma, favoring the propagation of microcracks when the material is under mechanical stress. However, in this work, the strict control of the O_2_ plasma conditions and the low time of exposure avoided a drastic damage of the PP fibers.

**Table 4:** Effect of the Low-Pressure O_2_ Plasma Treatment and PNIPAAm Grafting on the Bursting Properties OME and OMLP Meshes^a^

mesh	sample	burst force (N)	elongation to failure (mm)
OME	untreated	294.8 ± 11.50	12.9 ± 0.3
	plasma-treated	253.8 ± 13.30	11.5 ± 0.3
	OME-*g*-PNIPAAm	293.6 ± 17.40	12.7 ± 0.3
OMLP	untreated	218.2 ± 15.00	10.6 ± 0.1
	plasma-treated	218.9 ± 12.70	9.8 ± 0.03
	OMLP-*g*-PNIPAAm	245.8 ± 18.60	11.4 ± 0.6
	OMLP-*g*-PNIPAAm sterilized	257.2 ± 18.60	11.3 ± 0.3

a The effect of EtOx sterilization on grafted OMLP meshes is also displayed. The average value and the standard deviation of burst force and elongation to failure were obtained using five independent samples for each system.

On the other hand, the grafting of the PNIPAAm hydrogel on the plasma-treated meshes resulted in an improvement of the bursting properties for both knitting configurations. In particular, the burst force and the elongation at break determined for the OME-*g*-PNIPAAm mesh (293.6 ± 17.4 N and 12.7 ± 0.3 mm, respectively) were almost identical to those of untreated OME (294.8 ± 11.5 N and 12.9 ± 0.3 mm), indicating that the increment in the surface weight induced by the grafting process was enough to restore the damage produced by the O_2_ plasma treatment. In addition, the properties of OMLP-*g*-PNIPAAm experienced an advancement with respect to the pristine OMLP mesh (i.e., the burst force and the elongation at break increased by 13 and 7%, respectively). In this case, the enhancement of the surface weight was more advantageous in terms of improvements with respect to pristine samples due to the lightweight and the small damage of the plasma treatment in comparison to OME.

In order to evaluate if the beneficial effect of the grafted hydrogel increases with the surface weight, the burst properties of OME-*g*-PNIPAAm meshes with different surface weights (112.9 ± 22.3 and 185.2 ± 6.2 g/m^2^) were examined. The meshes with the highest surface weight were obtained using the procedure described in the Methods section but increasing the grafting time from 2 to 4 h. Figure S2, which compares the burst force and the elongation to failure, indicates similar values for the two samples. Hence, the burst force was 293.6 ± 17.40 and 292.8 ± 7.70 N for OME-*g*-PNIPAAm specimens with a surface weight of 112.9 ± 22.3 and 185.2 ± 6.2 g/m^2^, respectively, whereas the elongation at break was 12.6 ± 0.30 and 13.3 ± 0.03 mm, respectively. These values, which are clearly higher than those obtained for the plasma-treated mesh (Table 4), reflect that the beneficial effects provided by the grafting with PNIPAAm are practically independent of the surface weight and, therefore, of the mesh thickness increment (i.e., the thickness of untreated OME was 0.756 ± 0.007, increasing to 0.954 ± 0.211 and 1.096 ± 0.105 for OME-*g*-PNIPAAm with a surface weight of 112.9 ± 22.3 and 185.2 ± 6.2 g/m^2^, respectively).

**Figure 5:**
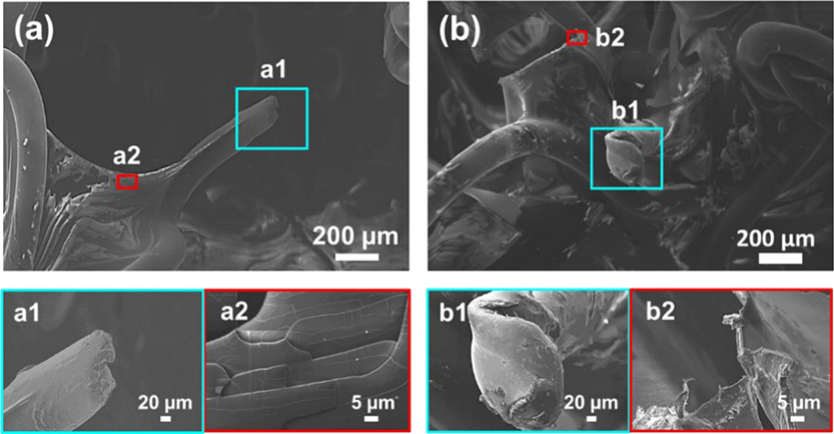
SEM micrographs of OME-*g*-PNIPAAm meshes with a surface weight of (a) 112.9 ± 22.3 and (b) 185.2 ± 6.2 g/m^2^ after rupture (up images). Magnified images show details of the fibers (a1 and b1) and the hydrogel coating (a2 and b2).

Figure 5 displays SEM micrographs of fractured OME-*g*-PNIPAAm meshes with the two studied surface weights after. Images show that the rupture of such coated meshes is very similar to that typically reported for fiber-reinforced hydrogel composites.^46,47^ On the other hand, comparison of the images recorded for the two meshes indicates that the deformation of the filaments increased with the amount of the grafted hydrogel (Figure 5a1–b1). Indeed, Figure 5b shows that the mesh with the highest surface density did not break at the point where the force was applied, which was attributed to the energy dissipation over the grafted hydrogel. Furthermore, the hydrogel coating exhibits superficial submicrometric defects (microcracks), even in the mesh with the lowest surface weight (Figure 5a2). Such microcracks may contribute to the mechanical failure of the mesh with the highest surface density, as suggests the propagations observed for OME-*g*-PNIPAAm with the highest surface weight (Figure 5b2).

Overall, results show that the stiffness of the meshes was significantly influenced by the grafting of PNIPAAm. The stiffness decreased for the grafted meshes, allowing the recovery of the mechanical performance shown by untreated samples (Figure 4). Among other factors, which are obviously connected to the soft consistency of the hydrogel, this improvement is related to the gel morphology on the PP yarns (Figure 5) and to the mesh architecture. It is worth noting that in order to mimic the environment of the final implantation, meshes were immersed at 37 °C for 15 min before the bursting tests. This feature, together with the amount of liquid absorbed by the grafted gel in its collapsed state, could explain the improved bursting properties since the mechanical integrity of PNIPAAm hydrogels was reported to be better in the collapsed state than in the swollen state.^48^ Another factor that should be considered is the effect of the polymerization temperature on the mechanical strength.^49^ In this work, the gel was polymerized at 30 °C, favoring a slow polymerization kinetics (i.e., formation of few polymer chains of high molecular weight) and, therefore, an improved strength.

### Suture Pull Out Forces of Untreated, Plasma-Treated, and Coated Meshes

**Figure 6:**
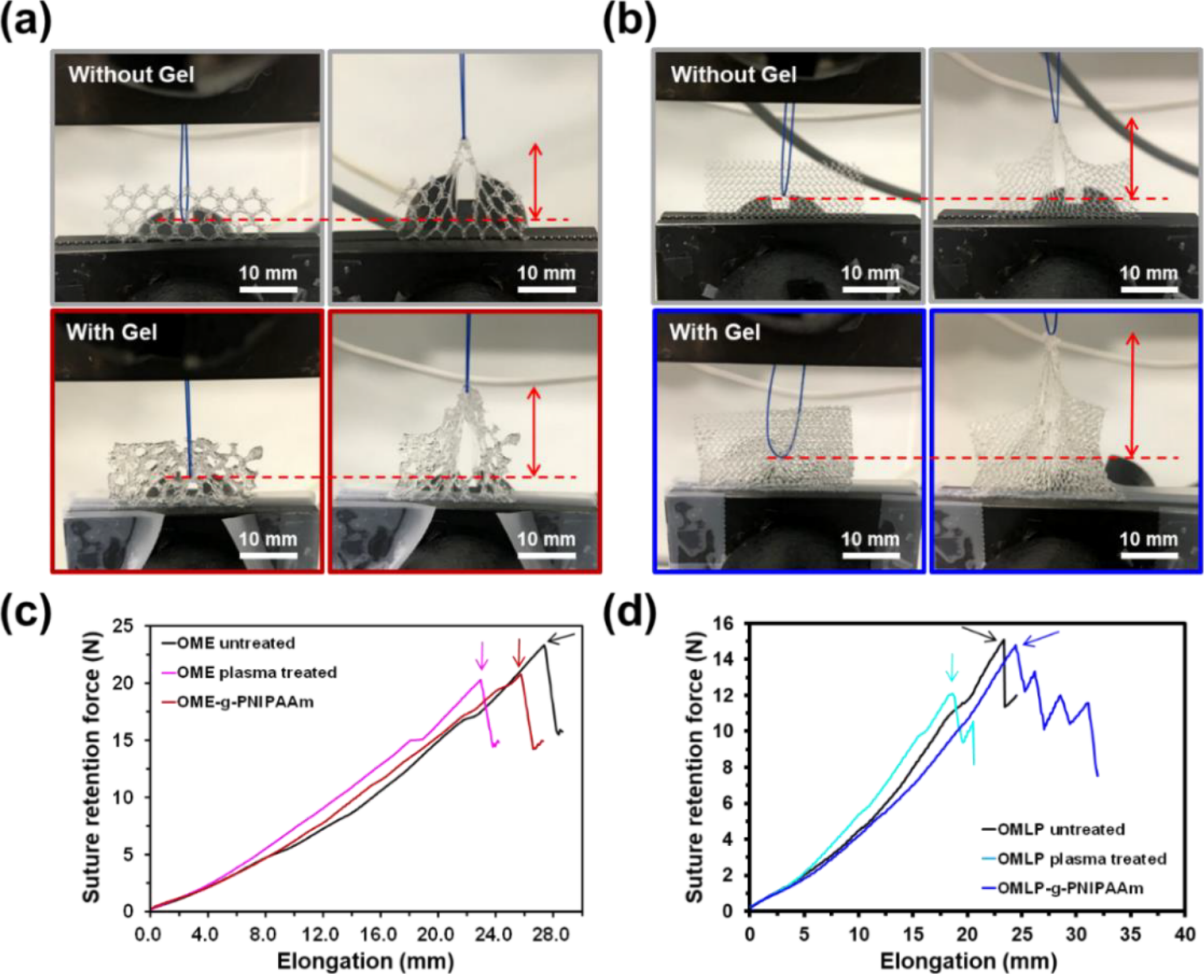
(a, b) Photographs of meshes subjected to suture pull out tests: (a) untreated OME (top) and OME-*g*-PNIPAAm (bottom) and (b) untreated OMLP (top) and OMLP-*g*-PNIPAA (bottom). (c, d) Suture retention versus elongation at failure curves corresponding to the breakage of the first filament: (c) untreated, plasma-treated, and hydrogel-modified OME meshes and (d) untreated, plasma-treated, and hydrogel-modified OMLP meshes. Arrows indicate the maximum strength values.

The effect of the mesh architecture, the plasma treatment, and the amount of the grafted hydrogel was investigated under suture retention forces employing one-point suture filament fixation. The procedure used to prepare the samples and to execute the suture pull out test is displayed in Figure S3. The purpose of such a test is to determine the maximum tension achievable before the separation between mesh and suture when they are pulled in opposite directions. However, note that the elongation and the pull out strength should not be considered precise metrics in this case since the suture may have captured different numbers of filaments to break through on different samples. Therefore, the results depend not only on the architecture of the mesh and the way the mesh is cut for analysis, but also on the tissue pattern into which the suture has been inserted. In order to minimize this limitation, herein, we focused our attention on the breakage of the first filament of the mesh material (Figure 6a,b). The suture pull out elongation curves are displayed in Figure 6c,d for pristine, plasma-treated, and hydrogel-modified meshes, while the resulting mechanical properties are listed in Table 5.

**Table 5:** Effect of the Low-Pressure O_2_ Plasma Treatment and PNIPAAm Grafting on the Suture Pull Out Forces of OME and OMLP Meshes^a^

mesh	sample	suture pull out force (N)	elongation (mm)
OME	untreated	*22.9 ± 0.7*	*27.4 ± 2.0*
	plasma-treated	20.3 ± 1.8	22.9 ± 1.2
	OME-*g*-PNIPAAm	20.5 ± 0.1	25.8 ± 1.9
OMLP	untreated	14.6 ± 0.7	24.3 ± 1.4
	plasma-treated	11.9 ± 1.8	18.9 ± 1.4
	OMLP-*g*-PNIPAAm	14.5 ± 1.6	24.5 ± 1.7
	OMLP-*g*-PNIPAAm sterilized	15.7 ± 1.3	27.8 ± 1.8

a The average value and the standard deviation of suture retention force and elongation were obtained using five independent samples for each system.

Untreated OME mesh exhibits higher suture pull out force and higher elongation at break (57 and 13%, respectively) than the untreated OMLP, which has been attributed to the elastic architecture of the former (i.e., pores are bigger in OME than in OMLP). For OME, plasma treatment reduced the suture pull out force and the elongation to failure of the first filament by only 11 and 16%, respectively. As occurred for the bursting properties, the addition of the PNIPAAm hydrogel to the plasma-treated PP fibers did not affect the material in terms of mechanical stability (Figure 6c,d and Table 5). Thus, OME-*g*-PNIPAAm exhibited properties that are intermediate between those of untreated and plasma-treated OME (i.e., the suture pull out force and the elongation at break of OME-*g*-PNIPAAm are smaller than those of untreated OME by only 10 and 6%, respectively).

Although the OMLP mesh was more sensitive to plasma treatment than the OME mesh, the plasma treatment did not cause substantial changes in the suture retention force or the elongation at break of the OMLP mesh. Thus, the suture pull out force and the first fiber elongation at break of plasma-treated OMLP were ∼20–22% smaller than those of the untreated mesh (Figure 6c,d and Table 5). In addition, the mechanical strength of OMLP-*g*-PNIPAAm was maintained almost unaltered in comparison to the pristine mesh. This observation demonstrates that the hydrogel was well bonded to the PP fibers of OMLP, as assumed in previous works.^22,23^

In order to investigate the effect of the amount of the grafted hydrogel on the suture pull out performance, additional assays were performed using the OME-*g*-PNIPAAm mesh produced with a grafting time of 4 h (i.e., a surface weight of 185.2 ± 6.2 g/m^2^). The suture retention strength and the elongation to the first filament failure for OME-*g*-PNIPAAm with the highest surface weight (Figure S4) were 23.5 ± 4.35 N and 34.9 ± 1.51 mm, respectively, indicating a higher performance than OME-*g*-PNIPAAm with the lowest surface weight (112.9 g/m^2^). This has been attributed to the hydrogel network morphology that, as shown above (Figure 5b), acts as a fiber reinforcement. In fact, changes in the pore diameter of the PP mesh due to the PNIPAAm coating are more important for suture retention than for bursting properties. In agreement with our results, Yu and Ma recently found that the suture retention strength of PP samples with small pore size is better than that of samples with larger pore size, whether in the warp or weft direction.^41^ Meshes with small pore size could withstand greater strength, even if they are subjected to significant curves and wrinkles. In this work, the pore size diameter of the mesh decreases in the presence of the hydrogel inducing greater strength and longer elongation, and this tendency increases with the amount of the grafted gel.^22^

### Effect of Temperature

**Figure 7:**
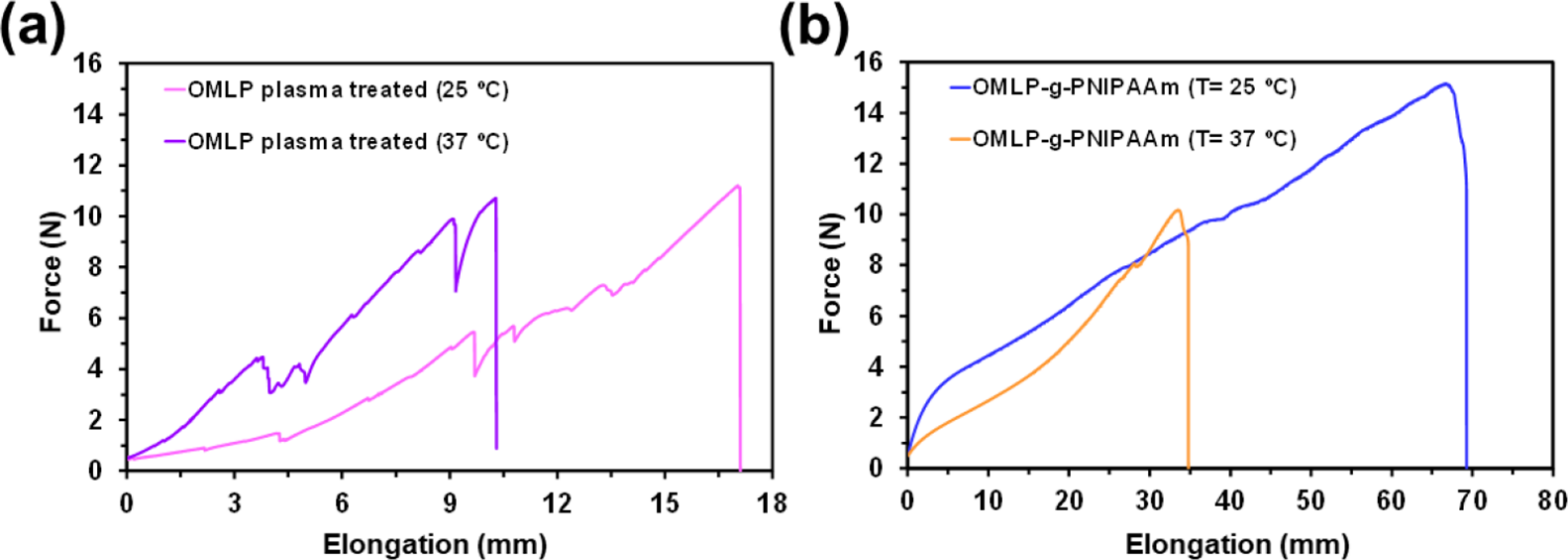
Strain–stress curves of (a) plasma-treated OMLP and (b) OMLP-*g*-PNIPAAm at 25 and 37 °C.

As the instruments used for bursting strength and suture pull out tests, which are specific for such assays, are not equipped with a temperature chamber, the tests presented in the previous subsections were carried out preheating the samples by immersion in distilled water at 37 °C for 15 min (i.e., according to the previously described ASTM method D-3787).^31^ However, in order to corroborate the beneficial effect of the hydrogel coating on the mechanical properties of the meshes, additional mechanical tests were carried out on plasma-treated OMLP and OMLP-*g*-PNIPAAm using a tensile-strain instrument equipped with a temperature chamber that allows the samples to be kept at constant temperature. Before carrying out the strain-deformation tests, the samples were kept at a temperature of 37 °C inside a chamber for 30 min. Results are shown in Figure 7 and Table 6.

**Table 6:** Mechanical Parameters Derived from Strain–Stress Curves for Plasma-Treated OMLP and (b) OMLP-*g*-PNIPAAm at 25 and 37 °C

sample	Young’s modulus (N/mm^2^)	force_max_ (N)	stroke_max_ (mm)	strain (%)
OMLP (25 °C)	3.0 ± 0.3	11.1 ± 1.6	15.7 ± 0.3	157 ± 7
OMLP (37 °C)	3.3 ± 0.5	10.7 ± 0.4	10.3 ± 0.6	103 ± 5
OMLP-*g*-PNIPAAm (25 °C)	2.7 ± 0.1	15.2 ± 2.0	33.5 ± 4.2	335 ± 18
OMLP-*g*-PNIPAAm (37 °C)	3.6 ± 0.3	10.2 ± 1.6	67.7 ± 6.3	667 ± 48

As it can be seen, the elastic modulus of plasma-treated OMLP is around 10% higher at the physiological temperature than at room temperature, while the strain decreases by 65% when the temperature increases from 25 to 37 °C. This last observation was attributed to the softening of the polypropylene threads that make up the mesh, which favored the unraveling of the mesh when applying tension. For this reason, the tests shown in this subsection should be considered only as a confirmation of the improvement provided by the grafted hydrogel. Thus, quantification of the mechanical properties of meshes must be performed through the bursting strength and suture pull assays, which were designed to avoid the fraying of the mesh fabric. On the other hand, inspection to the strain-deformation curves (Figure 7b) and the mechanical parameters (Table 6) obtained for OMLP-*g*-PNIPAAm obtained at 25 and 37 °C indicates that the Young modulus is higher at the latter than at the former, even though the fraying of the mesh was still observed. Furthermore, comparison between the mechanical parameters obtained for OMLP and OMLP-*g*-PNIPAAm evidences the significant improvement provided by the hydrogel, which is independent of the temperature.

### Mechanical Properties of Sterilized Untreated and Coated Meshes

**Figure 8:**
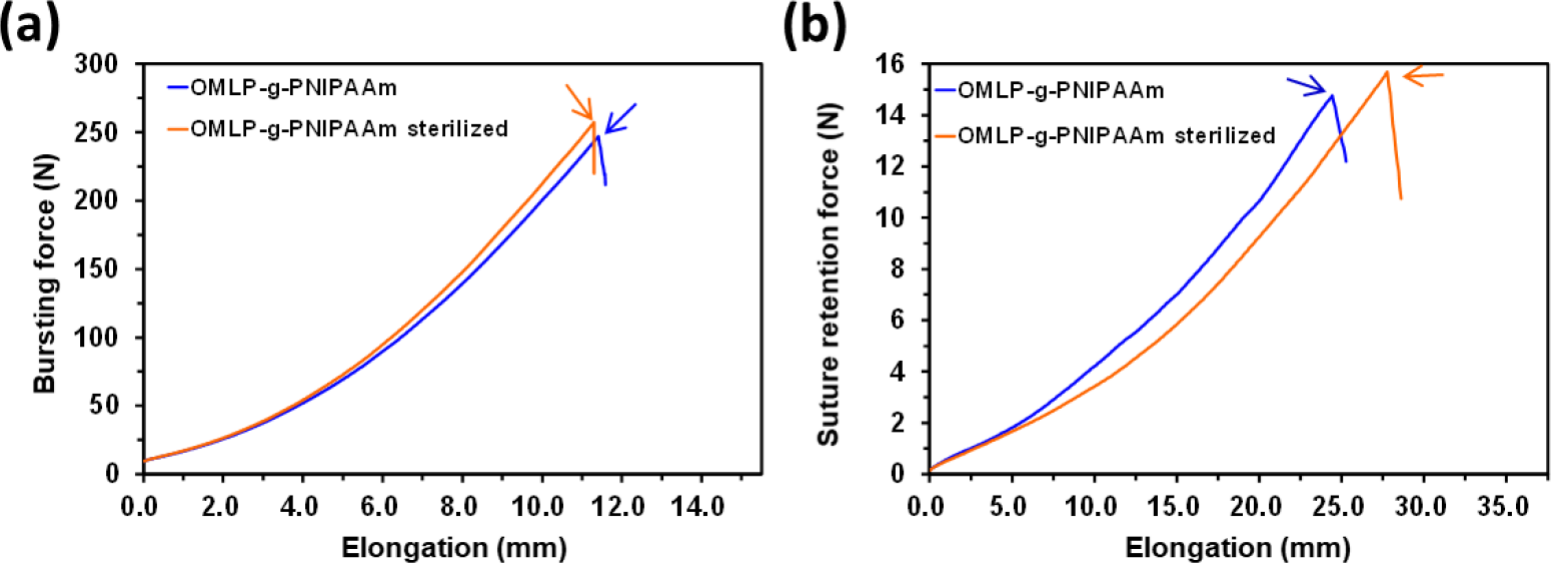
Effect of EtOx sterilization on the bursting and the suture pull out forces of OMLP-*g*-PNIPAAm. Arrows indicate the maximum strength values.

In order to study the effect of the EtOx sterilization process on the mechanical performance of the meshes grafted with the thermosensitive hydrogel, bursting and suture retention tests were carried out on sterilized OMLP-*g*-PNIPAAm samples (Figure 8). The OMLP mesh was chosen as the substrate since the small pore size and homogenous distribution of the hydrogel in OMLP-*g*-PNIPAAm resulted in a higher elongation than that achieved with OME-*g*-PNIPAAm. Furthermore, considering the demanding requirements that sterilization processes must meet (e.g., tolerance of the hernia mesh to the sterilization process, sterility assurance level, and residues of EtOx or other disinfectants^50^), both the sterility test and the evaluation of EtOx residues were performed on OMLP-g-PNIPAAm meshes using the ISO standards, as is described in the Methods section. The EtOx sterilization process did not change the surface weight and thickness of OME-*g*-PNIPAAm, as shown in Table 3.

The profiles displayed in Figure 8 indicate that the sterilization process has a moderate effect on the mechanical properties of the hydrogel-coated meshes. This is confirmed in Tables 4 and 5, which compare the values for the burst force, the suture pull out force, and the elongation to rupture before and after the process. More specifically, although the maximum burst and suture pull out forces were slightly higher for the sterilized samples than for the nonsterilized ones, such increment was of only 5 and 8%, respectively. In addition, the elongation was 13% for the sterilized sample than for the nonsterilized one (Figure 8b and Table 5). Amazingly, the EtOx cycle was performed at a temperature (∼ 40 °C) that is above the lower critical solution temperature (LCST) of PNIPAAm. Under such conditions, PNIPAAm chains usually contract due to the decreased amount of amide···water hydrogen bonds and to the increased amount of amide···amide hydrogen bonds, which explains the moderate increment in the burst and suture retention forces with respect to nonsterilized samples. Thus, the collapse of the hydrogel produced by the sterilization temperature seems to induce the behavior of PNPAAm as a plasticizer of the PP filaments, improving the resistance of the material to rupture.

**Figure 9:**
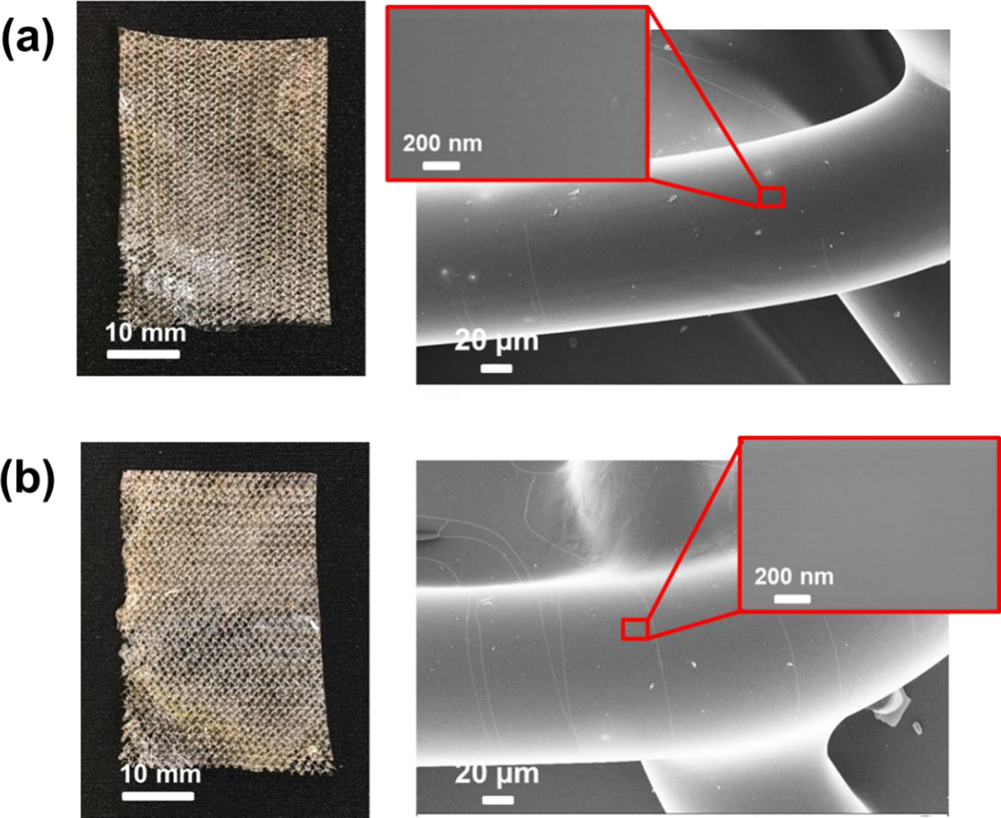
OMLP-*g*-PNIPAAm mesh (a) before and (b) after the sterilization with EtOx: optical image (left) and SEM micrographs (the inset corresponds to an 80k× magnified image of the region marked in the red box).

Optical images and SEM micrographs of OMLP-*g*-PNIPAAm before and after EtOx sterilization are displayed in Figure 9. Optical images show that the macroscopic textile structure is preserved after sterilization, while SEM micrographs evidence that the layer of the hydrogel coating the PP fibers was not significantly altered after the sterilization process. Thus, the hydrogel continues to be uniformly distributed and shows no damage compared to the nonsterilized sample. It should be mentioned that although sterilized samples exhibit a high amount of lineal cracks on the surface of the hydrogel (Figure 9b), such defects have not been attributed to the sterilization by itself but to the powerful vacuum system of SEM equipment. Indeed, mechanical tests performed on OMLP-*g*-PNIPAAm did not show appreciable differences with respect to those discussed above before sterilization.

**Figure 10:**
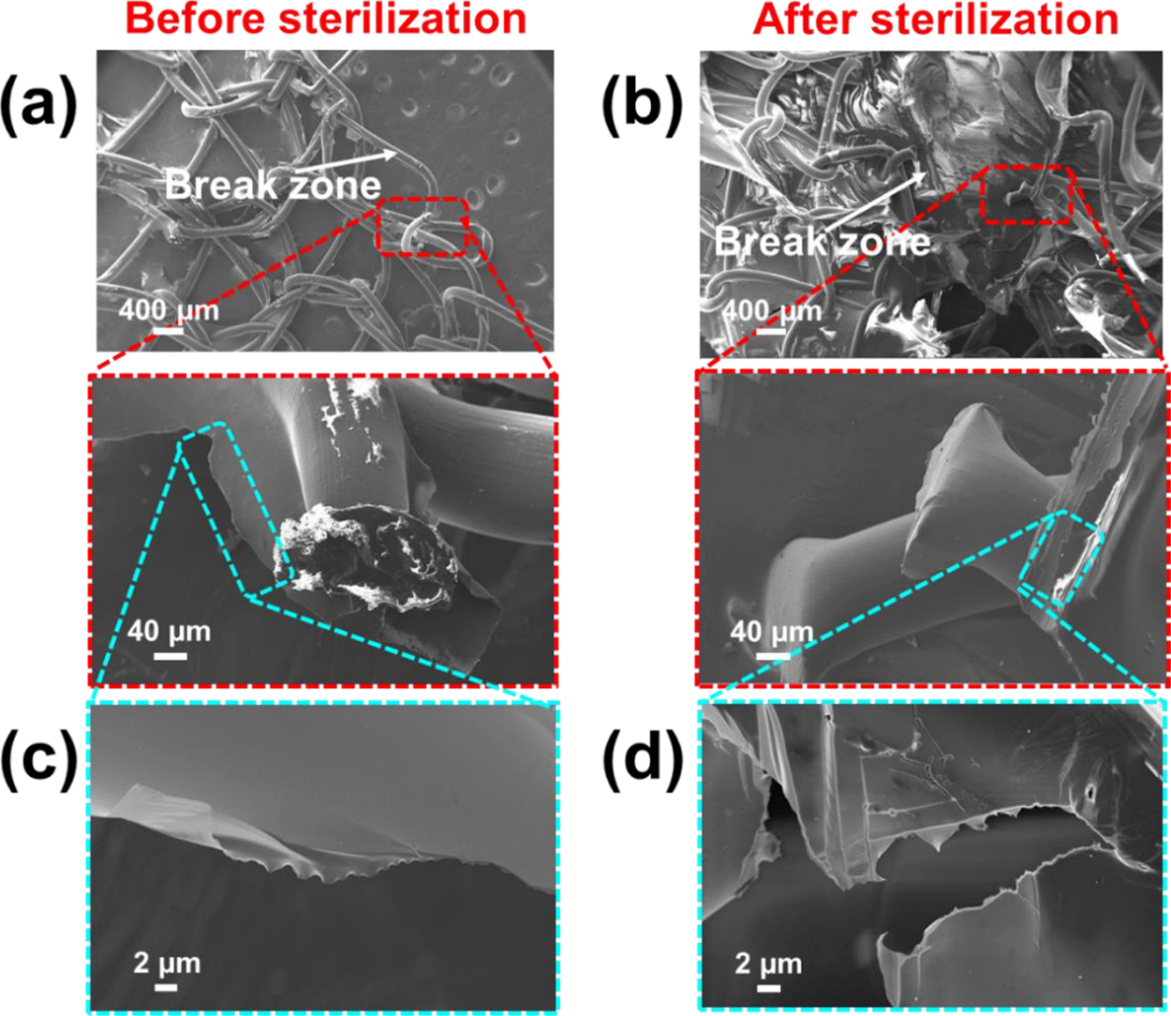
SEM micrographs of (a, c) nonsterilized and (b, d) sterilized OMLP-*g*-PNIPAAm meshes after break during the suture retention test: (a, b) Low and high magnification images of the fibers and (c, d) details of the grafted hydrogel. PP fibers and hydrogel coatings remain in a steady state after the sterilization process.

Inspection of SEM micrographs recorded for PP fibers broken during suture retention tests revealed some differences between nonsterilized and sterilized meshes (Figure 10). Nonsterilized specimens show a relatively smooth failure surface, sometimes appearing almost saw-cut and frequently displaying striations, representing cleavage steps or microbuckling caused by local flexural loading (Figure 10a). Conversely, sterilized specimens exhibit a highly ductile fracture that appears where the mesh is tightened to a single point (Figure 10b). This kind fracture usually results from high-strength failures in specimens without defects.^51^ On the other hand, Figure 9c,d shows details of the thin layer of the PNIPAAm hydrogel surrounding the PP fibers.

Evaluation of chemical residues coming from the EtOx sterilization process revealed the presence of 0.68 mg/unit of EtOx and of <0.5 mg/unit of ethylene chlorohydrin in sterilized OMLP-*g*-PNIPAAm meshes, such amounts decreasing to <10 μg/unit (EtOx) and <50 μg/unit (ethylene chlorohydrin) for the sterilized OMLP mesh (control). These results clearly show that the porous structure of the gel is able to entrap small amounts of EtOx residues if the process is performed at 40 °C. On the other hand, after 14 days of incubation of sterilized meshes in TSB medium, microbial growth remained absent in the culture vessels, proving again that sterilized OMLP-*g*-PNIPAAm meshes fulfill the high quality parameters demanded by this sterilization procedure.

## Conclusions

The potential utilization of a new generation of surgical meshes for hernia repair, which are prepared by grafting a thermosensitive hydrogel layer on commercial PP, has been examined by evaluating their mechanical performance through the different steps needed to implement such modification and after the ultimate sterilization process. Both bursting and suture retentions tests, which are normally conducted in industrial processes to assess the resistance and suitability of the meshes inside the physiological microenvironment after implantation, have been investigated in vitro. The plasma treatment, which was required for the successful grafting of the PNIPAAm hydrogel, induced a slight loss of mechanical properties, especially in OMLP meshes. However, the soft consistency of the coating hydrogel, as well as its arrangement above the PP fibers, led to enhanced elongations and better forces for both burst and suture retention tests. This beneficial effect was slightly more pronounced for OMLP meshes (lightweight) than for OME meshes (midweight).

On the other hand, results obtained after the EtOx sterilization process are favorable from the perspective of practical application. Thus, mechanical properties of the modified meshes were maintained and the mesh architecture was unaltered. In addition, the sterility tests and the evaluation of residues coming from the sterilization with EtOx were favorable. SEM studies concluded that the EtOx treatment produces a plasticizing effect.

Overall, comparison of the results from mechanical tests on unmodified meshes and those coated with a thin layer of PNIPAAm indicates that the hydrogel would be beneficial for the biomedical implant if it is subjected to abdominal wall forces after body implantation. Future investigations to complete the biocompatibility assessment and research under in vivo conditions will be necessary to validate the developed meshes as powerful biomedical implants.

## Supporting Information Available

Setup used for bursting and suture retention assays, procedure for the suture retention test, elongation vs bursting force and elongation vs suture retention force curves for the modified mesh with the highest hydrogel content (PDF)
