# Supplementary material for: Mechanical Properties of Smart Polypropylene Meshes: Effects of Mesh Architecture, Plasma Treatment, Thermosensitive Coating, and Sterilization Process
Source: ACS Biomater Sci Eng. 2023 May 26;9(6):3699–711. doi: 10.1021/acsbiomaterials.3c00311 (PMC10889589; doi:10.1021/acsbiomaterials.3c00311)
Supplement: Supplementary file 1 — ab3c00311_si_001.pdf [file ab3c00311_si_001.pdf]

SUPPORTING INFORMATION

Mechanical Properties of Smart Polypropylene Meshes: Effects of Mesh Architecture, Plasma Treatment, Thermosensitive Coating and Sterilization Process

Sonia Lanzalaco,[1,2,*], Christine Weis,[3] Kamelia A. Traeger,[3] Pau Turon,[3] Carlos Alemán,[1,2,4,*] and Elaine Armelin[1,2,*]

[1] IMEM-BRT Group, Departament d'Enginyeria Química, EEBE, Universitat Politècnica de Catalunya, C/ Eduard Maristany, 10-14, 08019, Barcelona, Spain

[2] Barcelona Research Center in Multiscale Science and Engineering, Universitat Politècnica de Catalunya, 08930 Barcelona, Spain

[4] Research and Development Centre, B. Braun Surgical, S.A.U. Carretera de Terrassa 121, 08191, Rubí, Barcelona, Spain.

[5] Institute for Bioengineering of Catalonia (IBEC), The Barcelona Institute of Science and Technology, Baldiri Reixac 10-12, 08028 Barcelona, Spain

E-mail: sonia.lanzalaco@upc.edu, carlos.aleman@upc.edu and elaine.armelin@upc.edu

Page S2	**Figure S1.** Set-up used to measure the mechanical properties of studied surgical meshes.<br>**Figure S2.** Effect of the surface weight of OME-*g*-PNIPAAm meshes on the bursting properties.
Page S3	**Figure S3.** Procedure used for the suture retention test.<br>**Figure S4.** Effect of the surface weight of OME-*g*-PNIPAAm meshes on suture pull out tests.

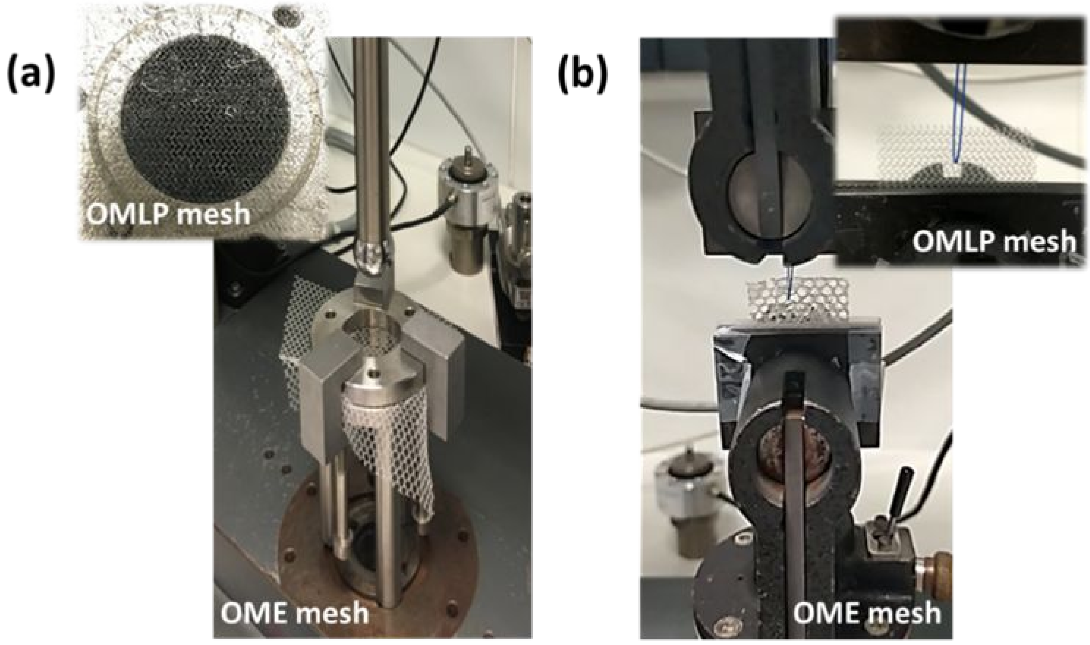

Figure S1. Set-up used to measure the mechanical properties of pristine, modified and sterilized surgical meshes: (a) bursting tests; and (b) suture pull out assays.

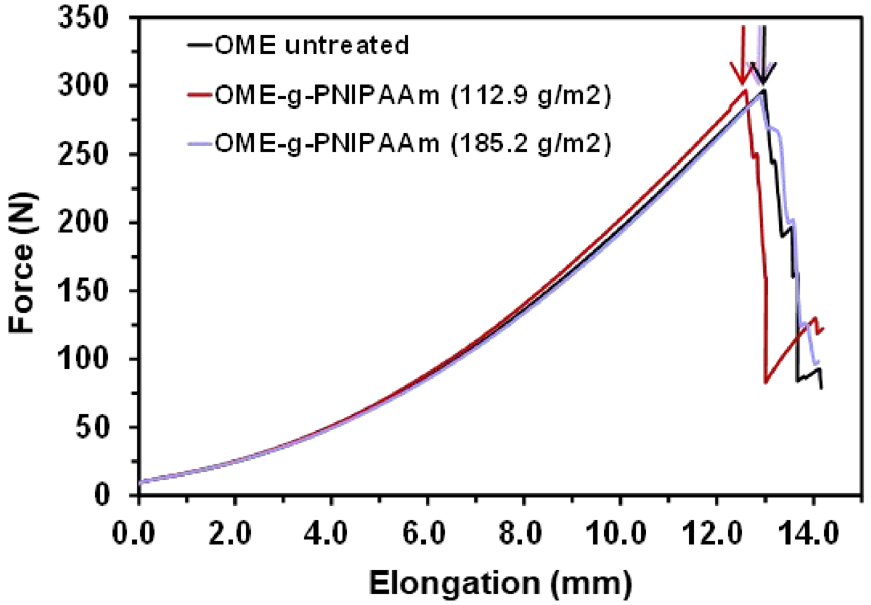

Figure S2. Effect of the surface weight of OME-*g*-PNIPAAm meshes on the bursting properties. Elongation *vs* force plots for untreated OME, OME-*g*-PNIPAAm with a surface weight of 112.9 ± 22.3 g/m^2 and OME-*g*-PNIPAAm with a surface weight of 185.2 ± 6.17 g/m^2 are displayed. Arrows indicate the maximum strength values.

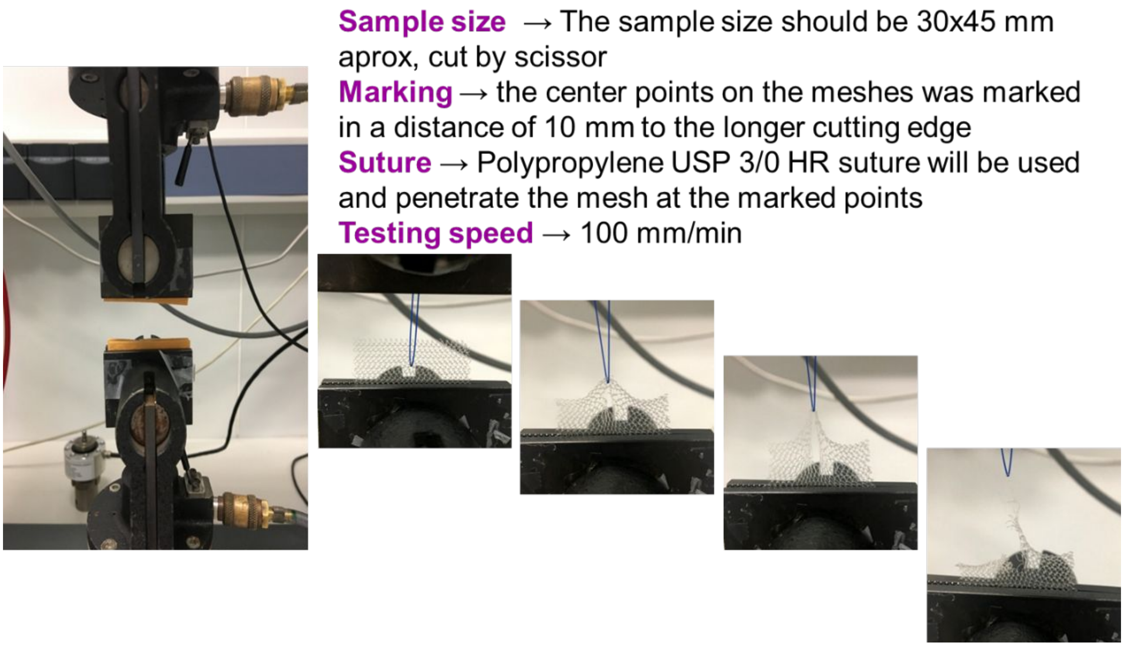

Figure S3. Suture retention test: Procedure used to prepare the sample and execute the assay.

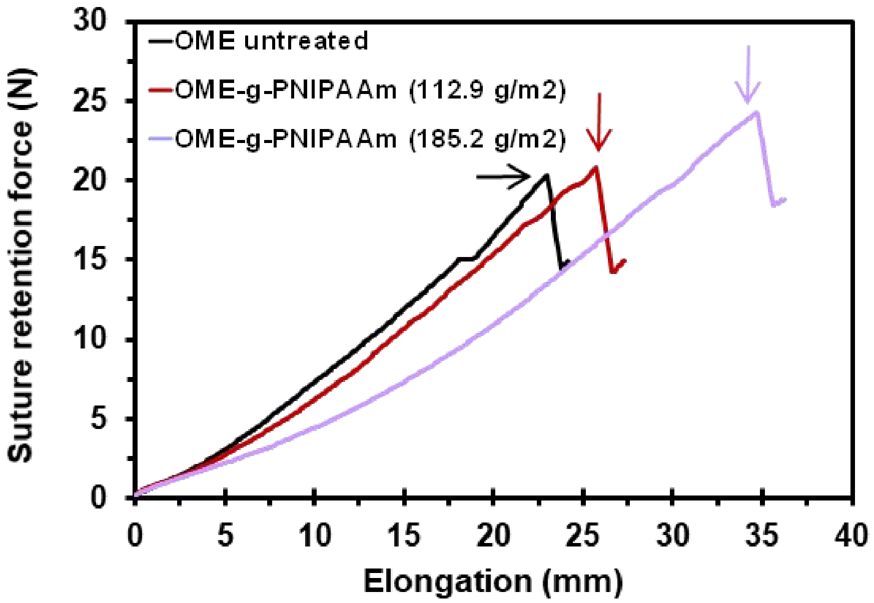

Figure S4. Effect of the surface weight of OME-*g*-PNIPAAm meshes on suture pull out tests. Elongation *vs* suture retention force plots of untreated OME, OME-*g*-PNIPAAm with a surface weight of 112.9 ± 22.3 g/m^2 and OME-*g*-PNIPAAm with a surface weight of 185.2 ± 6.17 g/m^2 are displayed. Arrows indicate the maximum strength values.
